# Subcutaneously Anchored Sutureless Device for Securement of Chest Tubes in Neonates with Pleural Effusion: Three Case Reports

**DOI:** 10.1155/2020/7480483

**Published:** 2020-03-10

**Authors:** Carmen Rodriguez Perez, Maria Grazia Romitti, Elena Pezzotti, Vito D'Andrea, Lucilla Pezza, Mauro Pittiruti

**Affiliations:** ^1^Neonatal Intensive Care Unit, ASST Spedali Civili, Brescia, Italy; ^2^Neonatal Intensive Care Unit, Fondazione Policlinico Universitario ‘A.Gemelli', Rome, Italy; ^3^Department of Surgery, Catholic University Medical School, Rome, Italy

## Abstract

We report the clinical cases of three neonates, all of them premature, requiring the placement of a chest tube for drainage of a massive pleural effusion. In all three patients, the chest tube was secured using a new subcutaneously anchored sutureless system. This new securement device was easy to insert and to remove, and highly effective in preventing dislodgment. Also, it was not associated to any undesired effect: no sign of pain and/or discomfort and no skin inflammation. The securement device proved to be comfortable and harmless even in fragile patients as neonates, including the frailest ones, the premature. To our best knowledge, this is the first report describing the use of such a device for this purpose.

## 1. Introduction

Massive pleural effusion is a rare but life-threatening condition in newborns, often requiring the insertion of a chest tube for pleural drainage, sometimes as an emergency procedure [1]. One of the most frequent complications of such a procedure is the early dislodgment of the tube due to failure of the methods commonly used for securement (adhesive skin systems/surgical stitches), resulting in an ineffective drainage of the pleural cavity and thus worsening of the patient's clinical conditions, requiring the placement of a novel chest tube. Every new placement of a chest tube is obviously associated with additional and potential risks for the patient, both inherent to the procedure itself and related to sedation.

## 2. Case Reports

We report the clinical cases of three neonates, all of them premature, requiring the placement of a chest tube for drainage of idiopathic congenital pleural effusion. In all three patients, chest tubes were secured using a new subcutaneously anchored sutureless device (SAS) (SecurAcath, Interrad).

### 2.1. Case 1

The first case was a male neonate born by urgent caesarean section (gestational age 32.2 weeks). The pregnancy had been complicated by polyamnios and massive bilateral hydrothorax of the fetus: the left pleural cavity had been drained by the gynecologists while the baby was still in the mother's womb. The weight at birth was 1970 g. The Apgar score was 1 in the first minute. Soon after birth, a right pleural effusion was evident at x-ray and ultrasound scan; 70 ml of pleural citrine liquid was drained from the right pleural space via a 21G intravenous cannula inserted into the second rib space at the midclavicular line. Because of severe respiratory distress, the patient was transferred to our Neonatal Intensive Care Unit (NICU); after that, tracheal intubation and mechanical ventilation with 100% oxygen was started. A chest X-ray showed a tension pneumothorax with almost complete atelectasis of the right lung and a left shift of the mediastinum, with compression of the left lung. Therefore, few hours after birth, an 8Fr pigtail catheter (Safe-T-Centesis Drainage System, BD) was placed into the right pleural cavity and secured with sterile strips (Steri-Strips, 3M): the mediastinum shifted back to its median line, with full expansion of the left lung. That same day, because of a massive pleural effusion, another pigtail catheter was also placed on the left side and secured with sterile strips; after only 36 hours, it was accidentally dislodged. On day 5, the right-side chest tube was still in place, but there was copious leakage from the insertion site; suspecting a misplacement, it was secured with surgical stitches; though, on day 7, we had to remove it because of malfunction.

**Figure 1:**
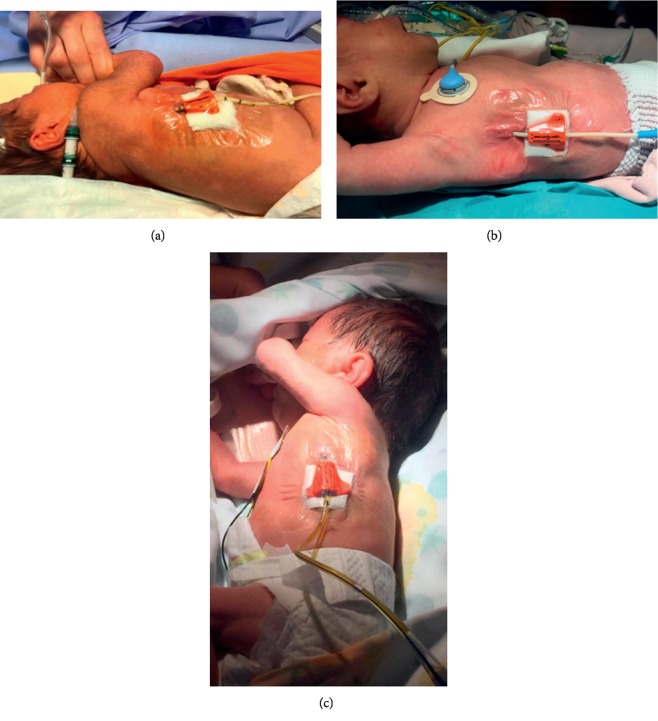
(a) Case 1: 8Fr pigtail catheter secured by SAS; the exit site is sealed with cyanoacrylate glue; a small piece of gauze is placed under the SAS device; the whole area is covered with transparent semipermeable membrane. (b) Case 2: same securement as in (a). (c) Case 3: 8Fr trocar catheter secured by SAS; the exit site is sealed with cyanoacrylate glue and covered with transparent membrane.

Two days later, on day 9, ultrasound scan showed a massive bilateral pleural effusion, so that 8Fr pigtail catheters were inserted again on both sides and secured with sterile strips. On day 21, the left-side catheter was completely dislodged; the right-side catheter was progressively dislodged in the following days, so that on day 23 half of the length of the catheter was out of the thorax. In order to avoid complete dislodgement, we decided to remove the sterile strips and anchor the catheter with an 8Fr SAS; the exit site of the catheter was sealed with sterile cyanoacrylate glue and covered with transparent semipermeable dressing (Figure 1(a)). The device was well tolerated; no sign of discomfort and/or pain was detected, and no manifestation of skin irritation or inflammation was noticed. The right-side chest tube stayed in place, well-functioning for ten days, and was eventually removed on day 33, since pleural drainage was not necessary any longer. After appropriate medical treatment (parenteral nutrition, milk formula enriched with medium chain triglycerides, intravenous infusion of octreotide, and a somatostatin analog), the patient was eventually discharged on day 72 in good general condition with regular weight increase, feeding on mother's milk.

### 2.2. Case 2

This female neonate was born at 34.2 weeks of gestational age by urgent caesarean section, due to complicated biamniotic-bicorial twin pregnancy with ascites effusion detected by intrauterine ultrasound scan at week 33. Before the delivery, a fetal thoracentesis had been performed on the left hemithorax. The weight at birth was 2,330 g. The Apgar score was 6 on the first minute of life. In the delivery room, 20 ml of citrine fluid was drained from the left hemithorax inserting directly a 21G butterfly needle under ultrasound guidance, using a wireless linear probe (Linear wireless probe 256 HF, ATL). Immediately after birth, the baby was intubated and admitted to our NICU on mechanical ventilation. An ultrasound scan revealed a hydrothorax also on the right side (that same side that had been drained while inside the womb): an 8Fr pigtail catheter was placed for pleural drainage under ultrasound guidance using the wireless linear probe and anchored with an 8Fr SAS. The exit site was sealed with sterile cyanoacrylate glue and covered with transparent semipermeable dressing (Figure 1(b)). On day 33, because of failure of medical treatment (total parenteral nutrition and intravenous infusion of octreotide), a thoracoscopy was performed so to ligate the thoracic duct. During the procedure, the 8Fr catheter, which had stayed in place anchored with the SAS for 32 days without complications, was removed and replaced with a 10Fr tube (Trocar Thoracic Catheter, Redax) that was secured by surgical stitches. Due to the worsening of the baby's condition, a CT scan revealed an abundant persistent pouch of pleural fluid on the right side, causing the dislocation of the mediastinum and compression of the right inferior lung lobe. On day 37, a new 8Fr pigtail catheter was placed to drain the major anteroposterior pouch of fluid, secured with sterile strips, and covered with transparent semipermeable dressing. On day 38, a pleurodesis was performed on the right side, by introducing 4% iodine-povidone through the chest tube. This had a positive effect, so that on day 45 both thoracic catheters were removed. The patient's conditions improved, and the patient was eventually discharged on day 76.

### 2.3. Case 3

The third case was a male neonate born by urgent caesarean section (gestational age 32 weeks) with a prenatal history of polyamnios and fetal massive bilateral hydrothorax. Two thoracenteses had been performed in uterus; chylothorax diagnosis had been confirmed by the analysis of the pleural fluid. At delivery, because of persistent respiratory distress, the child was intubated and transferred to our Neonatal Intensive Care Unit. The Apgar score at 1 and 5 minutes was 7–9 and the birth weight was 1970 g. An ultrasound scan showed a massive pleural effusion in the left pleural space. A 21G intravenous cannula was inserted into the fifth rib space just behind the anterior axillary line, and 110 ml of citrine fluid was drained, with immediate clinical improvement. Afterward, chest ultrasound was daily performed to verify the magnitude of the effusion. At day 15, a relevant pleural effusion was detected in the left pleural space. At day 17, the clinical conditions worsened, and an 8Fr catheter (Trocar) was placed into the pleural space under ultrasound guidance, anchored with an 8Fr SAS; the exit site of the catheter was sealed with sterile cyanoacrylate glue and covered with transparent semipermeable dressing (Figure 1(c)). This chest tube remained in place for 12 days, always well-functioning. It was removed on day 29. Chylothorax was progressively reduced by medical treatment (octreotide and medium chain triglycerides formula). The infant was eventually discharged in good health on day 50.

## 3. Discussion

The placement of a pleural drainage is an invasive procedure, since it implies piercing the chest wall and introducing a large bore catheter into the pleural cavity [1]. The maneuver is associated with both immediate complications such as local hemorrhage, pneumothorax, or hemothorax—which may be minimized by the adoption of ultrasound guidance—and late complications such as local infection or dislodgment of the catheter. The latter is particularly frequent; incomplete dislodgment often yields malfunction of the drainage, while complete dislodgment implies the need of repositioning the catheter, with relevant impact on morbidity and health costs.

Current strategies of securement of chest tubes in neonates are far to be satisfactory. Securement by cutaneous suture is traumatic, potentially associated with local skin infection, and ultimately ineffective. In this case report, we used securement by stitches in the first neonate, trying to save a partially dislodged pigtail catheter, but this type of securement was ineffective: the catheter was completely dislodged after 2 days.

**Figure 2:**
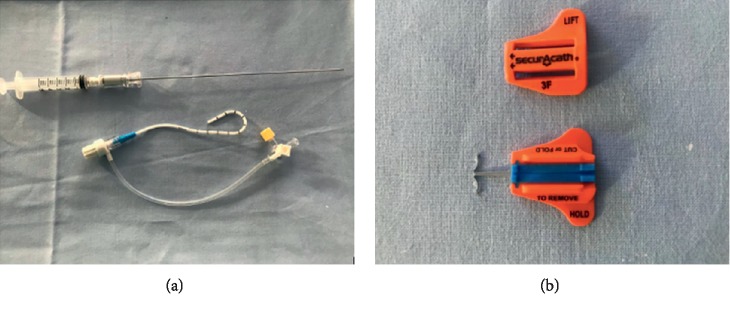
(a) 8Fr pigtail catheter (Safe-T-Centesis Drainage System, BD), used in cases 1 and 2. (b) Subcutaneously anchored system (SecurAcath, Interrad). Before use, the system consists of two components: the anchor base and the cover.

Chest tubes with pigtail end are now widely used, also in our NICUs: their peculiar shape is designed to reduce the risk of accidental dislocation of the catheter (Figure 2(a)). Though they should not strictly require external securement, we often secure them by adhesive sterile tapes and transparent semipermeable dressing. In our first case, four pigtails secured with sterile strips were not particularly effective: one catheter was lost after 36 hours, another required securement by suture after 5 days because of partial dislodgment, another one dislodged completely after 12 days, and a fourth was partially dislodged after 14 days.

As reported by these three cases, we are currently testing an innovative approach to chest tube securement, using a new subcutaneously anchored sutureless device (SAS) (SecurAcath, Interrad) initially marketed for the securement of central venous catheters. Its safety, efficacy, and cost effectiveness are well acknowledged in the world of vascular access [2–5]. SAS has also been used for other purposes: for instance, as securement of cerebral-spinal catheters [6], biliary tube drainage, and nephrostomy tubes [7]. To our best knowledge, there are no published reports about the use of SAS to anchor thoracic catheter for pleural drainage and no reports at all about its use for securement of chest tubes in neonates. In our first experience, reported in this paper, SAS was highly effective and safe. In the first case, a pigtail catheter was kept in place by SAS for 10 days without any undesired effect: the catheter was removed electively and not because of malfunction or dislodgment. In the second neonate, a pigtail catheter was secured with SAS and stayed in place for 32 days without any complication: the catheter was eventually removed because it was not necessary anymore. In the third neonate, an 8Fr trocar thoracic tube—secured with SAS—stayed in place without any complication for 12 days.

Some considerations about insertion and management of the SAS may be interesting.

First, we must consider that different SAS are available for different size of catheters. In fact, the design of SAS is such that, while the anchor blocks the device under the skin, the catheter itself is blocked by the two portions of the device (Figure 2(b)), inside a special groove that must be of appropriate size. In other words, 8Fr tubes require 8Fr SAS, 10Fr tubes require 10Fr SAS, and so on.

Also, we noticed that insertion of SAS by anchoring it in the subcutaneous tissue was extremely easy. As suggested by our previous experience with SAS for securement of central venous catheters in neonates, we placed a small piece of sterile gauze under the base of the device so to avoid the risk of an excessive pressure of the device over the delicate skin [2].

Furthermore, the entrance of the SAS and of the chest tube through the skin was sealed by a few drops of cyanoacrylate glue; though this strategy may add little or nothing in terms of securement, our experience with venous catheters suggest that it is very effective in blocking any oozing or bleeding from the breech.

As regards the maintenance, the securement with SAS allowed an optimal periodic disinfection of the skin around the chest tube, which is difficult or impossible when using stitches or sterile strips. As already described, no skin abnormalities of any kind were observed: no inflammation, no irritation, and no bleeding. Also, no sign of local pain or discomfort was evident in any of the three neonates whose chest tube was secured by SAS.

In conclusion, we recommend SAS as a safe and effective alternative option for securing chest tubes in neonate: it is easy to insert and easy to remove; it is not associated with any undesired effect, not even in premature newborns; most of all, it minimizes and virtually eliminates the risk of accidental dislodgment of the chest tube, an event associated with increased morbidity and increased health cost.

Though we report a very initial clinical experience, we think that our clinical results suggest the interest to conduct further studies about the use of SAS in this clinical situation.
